# Educational attainment mitigates cognitive decline associated with elevated pTau181 and APOE ε4 in Puerto Ricans

**DOI:** 10.1002/alz70860_104908

**Published:** 2025-12-23

**Authors:** Daniel A. Dorfsman, Dingtian Cai, Kara L Hamilton‐Nelson, Larry D Adams, Pedro R Mena, Katrina Celis, Vanessa C. Rodriguez, Jose J. Sanchez, Glenies S. Valladares, Mariangelie Lopez, Patrice G Whitehead, Michael B. Prough, Sabrina M. Mas, Carolina Scaramutti, Heriberto Acosta, Concepcion Silva‐Vergara, Katalina F. McInerney, Anthony J Griswold, Briseida E. Feliciano‐Astacio, Michael L Cuccaro, Jeffery M Vance, Azizi A Seixas, Margaret Pericak‐Vance, Farid Rajabli

**Affiliations:** ^1^ John P. Hussman Institute for Human Genomics, University of Miami Miller School of Medicine, Miami, FL, USA; ^2^ Dr. John T. Macdonald Foundation Department of Human Genetics, University of Miami Miller School of Medicine, Miami, FL, USA; ^3^ University of Miami Miller School of Medicine, Miami, FL, USA; ^4^ Department of Informatics and Health Data Science, University of Miami Miller School of Medicine, Miami, FL, USA; ^5^ Department of Psychiatry and Behavioral Sciences, University of Miami Miller School of Medicine, Miami, FL, USA; ^6^ Clinica de la Memoria, San Juan, PR, USA; ^7^ Universidad Central del Caribe, Bayamón, PR, USA; ^8^ Department of Neurology, University of Miami Miller School of Medicine, Miami, FL, USA

## Abstract

**Background:**

Educational attainment (EA) is a key contributor towards cognitive reserve, providing the brain with a degree of resilience to Alzheimer's disease pathology (ADP). However, research suggests that the influence of education on cognitive reserve varies across racial and ethnic groups and *APOE* genotype, highlighting the importance of characterizing these effects in diverse populations. This study explores the relationship between EA, *APOE* ε4 status, and cognitive function in a Puerto Rican (PR) cohort, focusing on individuals exhibiting elevated ADP as indicated by plasma pTau181 levels.

**Method:**

A subset of 793 PR older adults with elevated (>mean+1SD, *n* = 124) plasma log(pTau181) was analyzed. Cognitive function was estimated using a composite functional score calculated as the sum of the non‐memory items in the Clinical Dementia Rating scale (CDR‐FUNC, range=0‐12). EA was dichotomized as high (>9 years) and low (≤9 years). Associations between CDR‐FUNC, EA, and *APOE* ε4 carrier status were performed using the Mann‐Whitney U test.

**Result:**

Among individuals with elevated pTau181, those with lower EA exhibited significantly worse cognitive function (*p* = 6.5×10^‐4^). When stratified by *APOE* ε4 status, the association between EA and cognitive function was more pronounced among ε4 carriers (*p* = 1.52×10^‐3^) than non‐carriers (*p* = 0.06). In addition, within the low EA group, *APOE* ε4 carriers had poorer cognitive function than non‐carriers (*p* = 0.045). However, this difference was not observed in the high EA stratum.

**Conclusion:**

These results suggest that EA may enhance cognitive reserve and promote resilience to ADP in the PR population. Overall, high EA confers a cognitive advantage against functional decline in the presence of elevated pTau181. Furthermore, carrying the *APOE* ε4 allele increases the risk of cognitive decline in individuals with low EA compared to those with high EA. These findings underscore the importance of considering both educational background and genetic risk factors in efforts to understand and develop interventions to prevent cognitive decline globally.

